# Supplementary material for: Mammo-AGE: deep learning estimation of breast age from mammograms
Source: Nat Commun. 2025 Dec 8;16:10934. doi: 10.1038/s41467-025-65923-5 (PMC12686398; doi:10.1038/s41467-025-65923-5)
Supplement: Supplementary file 1 — Supplementary Information [file 41467_2025_65923_MOESM1_ESM.pdf]

Supplementary Materials for

Mammo-AGE: Deep Learning Estimation of Breast Age from Mammograms

Xin Wang, Tao Tan,* Yuan Gao, Hong-Yu Zhou, Tianyu Zhang, Luyi Han, Eric Marcus, Chunyao Lu, Caroline A. Drukker, Jonas Teuwen, Regina Beets-Tan, Shanshan Wang, Nico Karssemeijer, Ritse Mann

*Corresponding author. Email: taotanjs@gmail.com

This PDF file includes:

Supplementary Fig.1 - 13
Supplementary Table 1 - 9

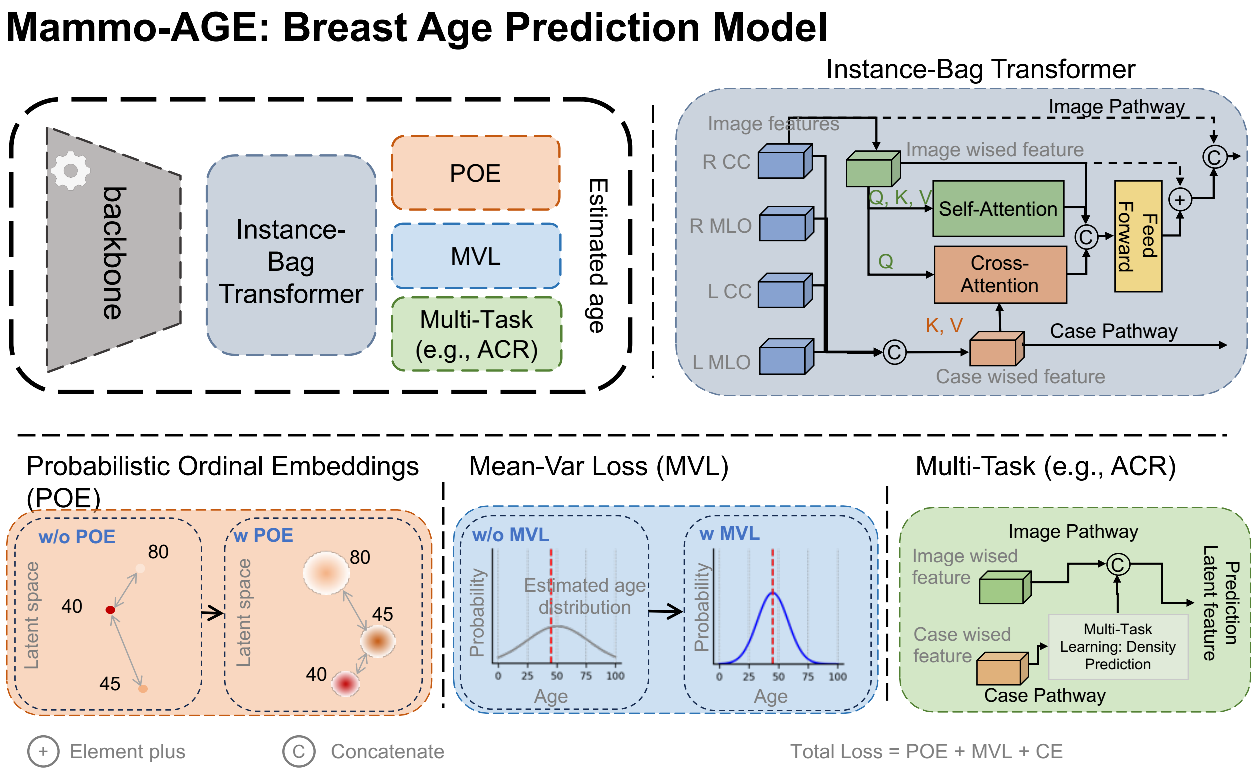

Supplementary Fig. 1. Detailed description of the network architecture. The model is designed to learn from mammograms and predict the chronological age of women, termed "breast age". By incorporating information from multi-view mammograms rather than using a single view, the model can enhance its ability to capture features related to breast aging. To achieve this, we draw inspiration from the global-local transformer (GLT) framework proposed by [1] and introduce an instance-bag transformer. This transformer consists of self-attention and cross-attention blocks, facilitating the integration of information from multiple views. Q, K, and V in the Instance-Bag Transformer refer to the query (Q), key (K), and value (V) matrices used in the attention mechanisms to capture relationships between instances (single views) and bags (four-view examination). The model incorporates multi-task learning for density prediction and uses a combination of cross-entropy (CE) loss, mean-variance (MVL) loss [2], and probabilistic ordinal embedding (POE) loss [3] functions to constrain the learning process. Five different backbone-based (ResNet-18, ResNet-50, ConvNeXt-Tiny, EfficientNet-B0, and DenseNet-121) models were ensembled by weighted averaging of predicted ages.

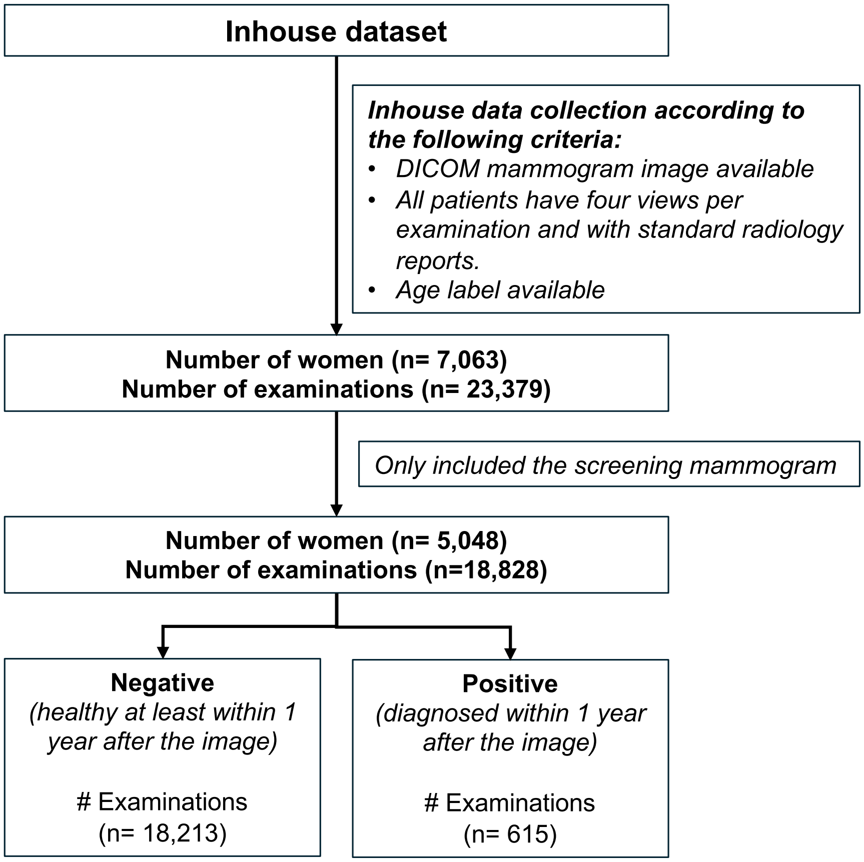

Supplementary Fig. 2. The flowchart of the inhouse dataset collection.

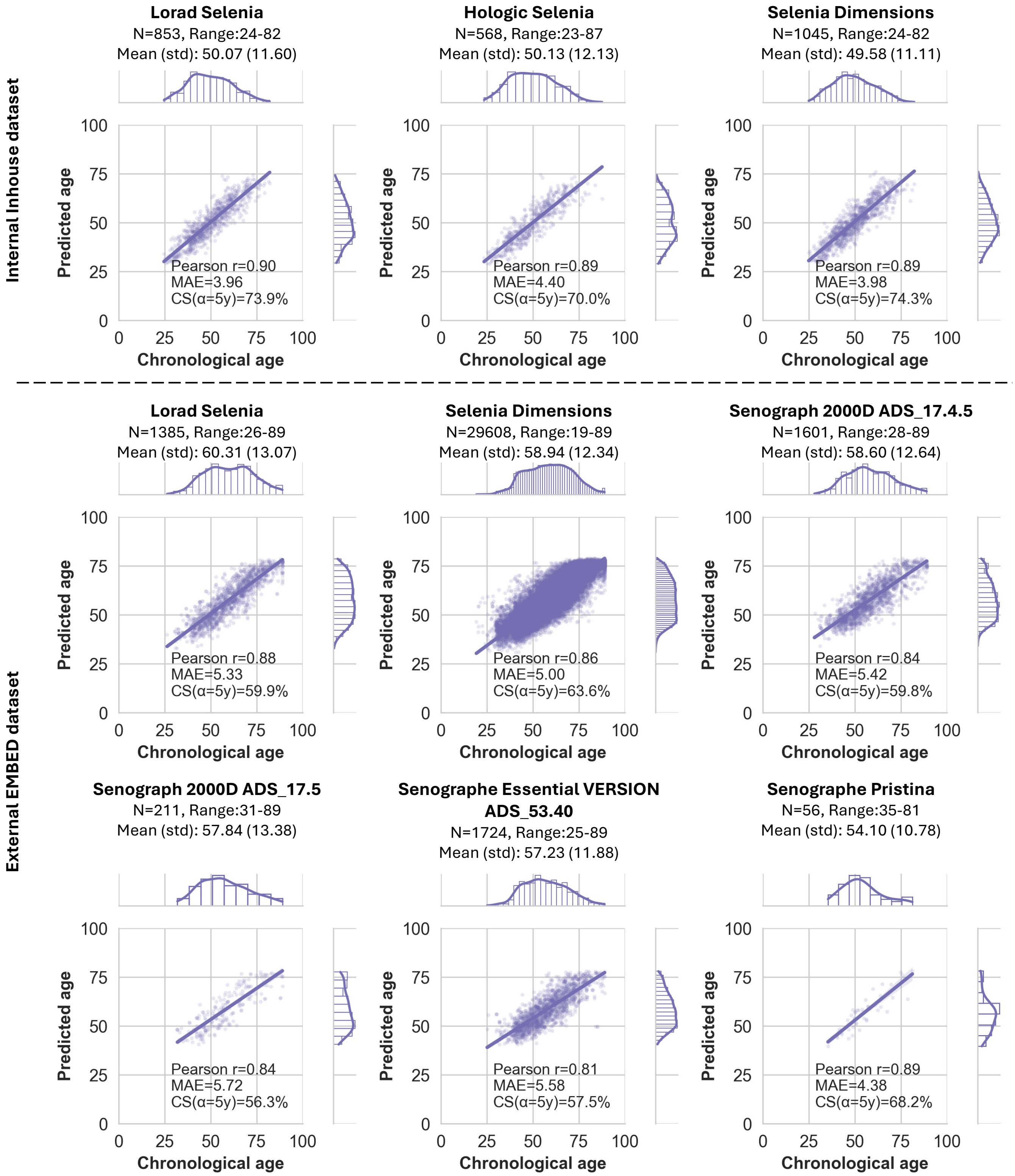

Supplementary Fig. 3. Our model's performance based on different scanners across both Inhouse and external EMBED datasets.

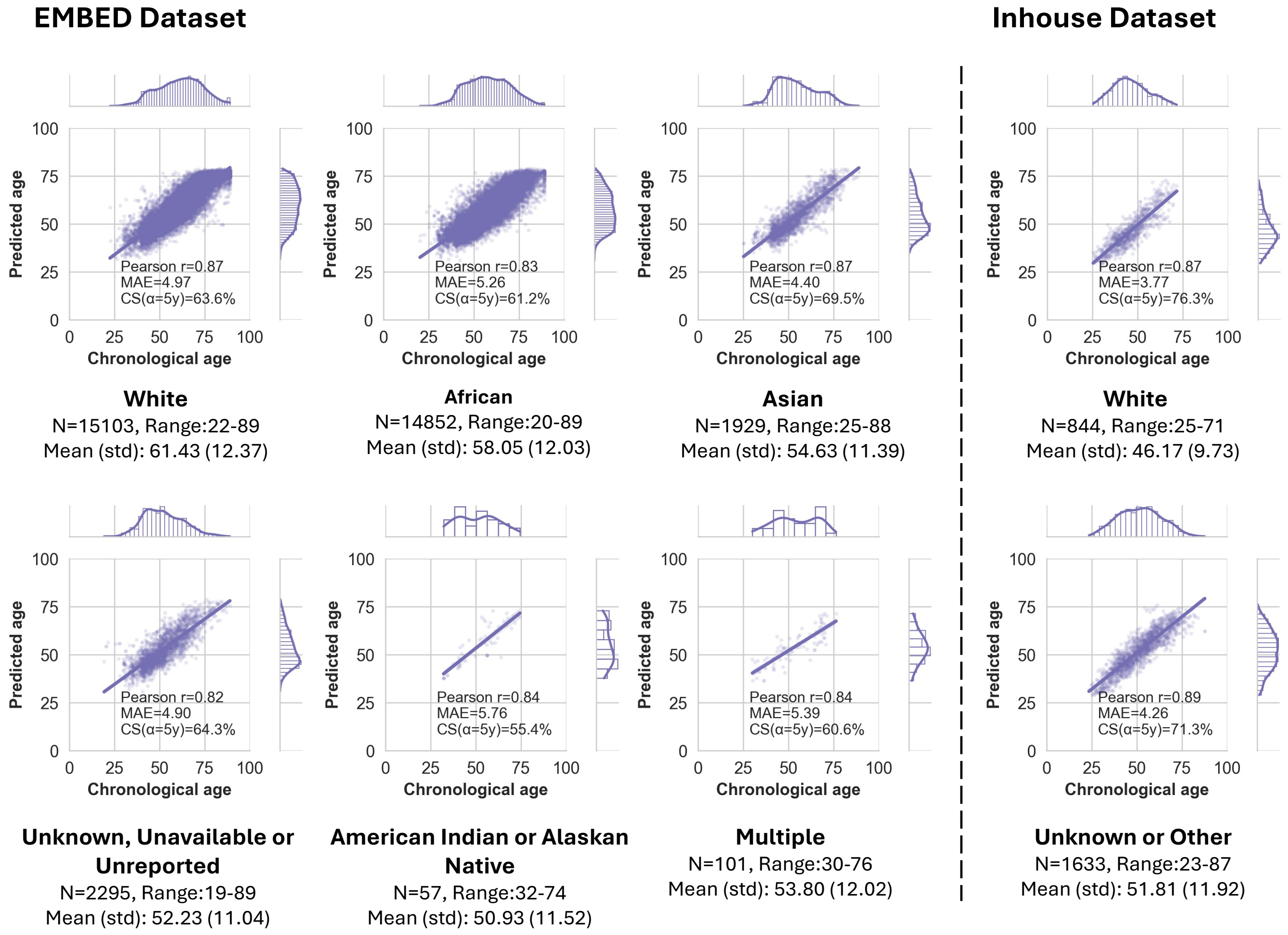

Supplementary Fig. 4. Our model's performance across different race subgroups in both inhouse and external EMBED datasets.

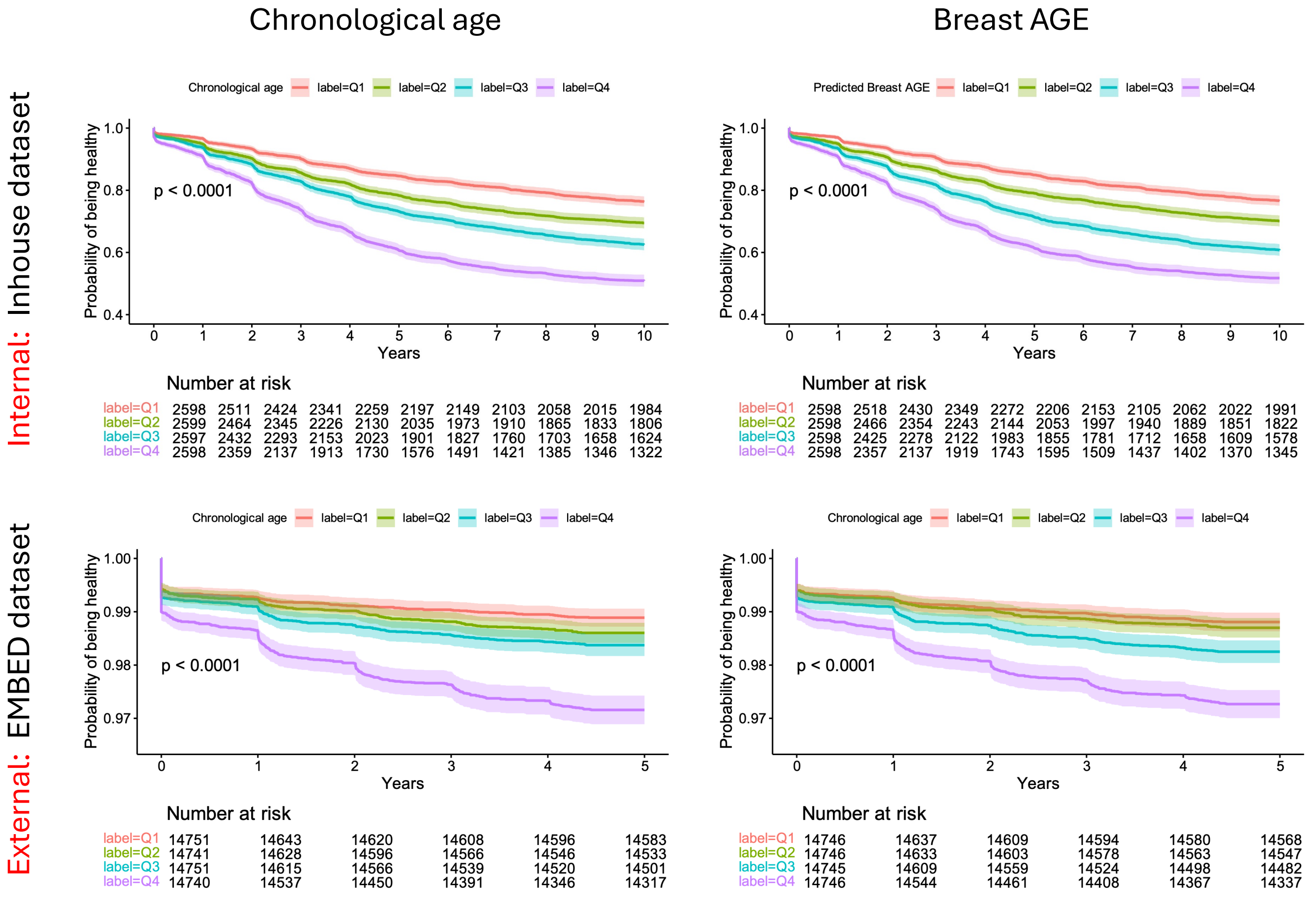

Supplementary Fig. 5. Kaplan–Meier estimates of breast cancer risks by chronological age and predicted breast age in inhouse and external EMBED datasets. Note: In calculating these Kaplan-Meier curves, breast ages were predicted from mammograms and have not been adjusted for chronological age. Therefore, the results indicate that breast age may serve as a similar risk factor for breast cancer as chronological age. Statistical significances were calculated using a two-sided log-rank test. Shaded areas represent the 95% confidence intervals.

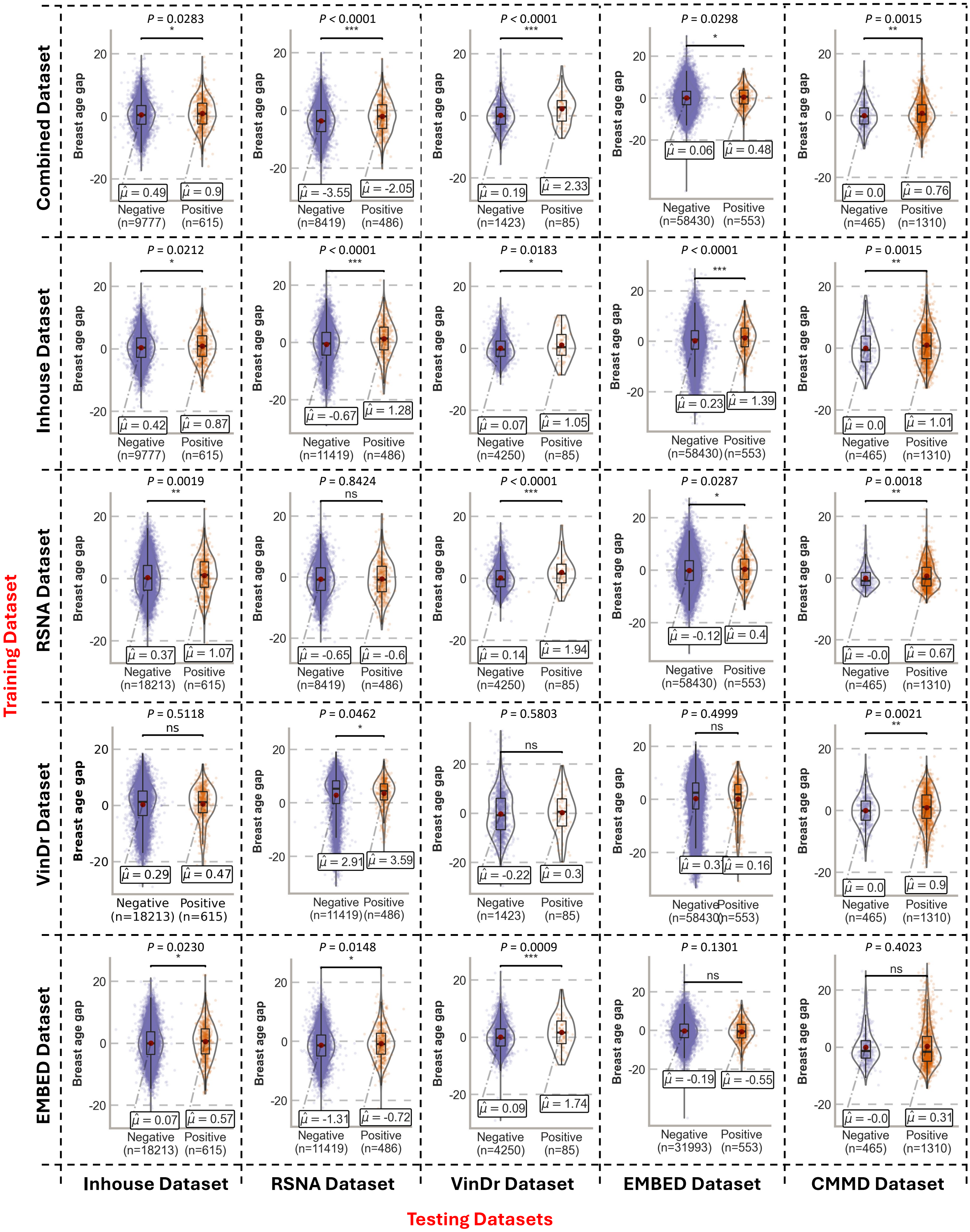

Training Dataset
Combined Dataset
Inhouse Dataset
RSNA Dataset
VinDr Dataset
EMBED Dataset
Testing Datasets
Inhouse Dataset
RSNA Dataset
VinDr Dataset
EMBED Dataset
CMMD Dataset
Breast age gap
20
0
-20
Negative
Positive
P = 0.0283 *
μ̂ = 0.49 μ̂ = 0.9
Negative (n=9777) Positive (n=615)
P < 0.0001 ***
μ̂ = -3.55 μ̂ = -2.05
Negative (n=8419) Positive (n=486)
P < 0.0001 ***
μ̂ = 0.19 μ̂ = 2.33
Negative (n=1423) Positive (n=85)
P = 0.0298 *
μ̂ = 0.06 μ̂ = 0.48
Negative (n=58430) Positive (n=553)
P = 0.0015 **
μ̂ = 0.0 μ̂ = 0.76
Negative (n=465) Positive (n=1310)
P = 0.0212 *
μ̂ = 0.42 μ̂ = 0.87
Negative (n=9777) Positive (n=615)
P < 0.0001 ***
μ̂ = -0.67 μ̂ = 1.28
Negative (n=11419) Positive (n=486)
P = 0.0183 *
μ̂ = 0.07 μ̂ = 1.05
Negative (n=4250) Positive (n=85)
P < 0.0001 ***
μ̂ = 0.23 μ̂ = 1.39
Negative (n=58430) Positive (n=553)
P = 0.0015 **
μ̂ = 0.0 μ̂ = 1.01
Negative (n=465) Positive (n=1310)
P = 0.0019 **
μ̂ = 0.37 μ̂ = 1.07
Negative (n=18213) Positive (n=615)
P = 0.8424 ns
μ̂ = -0.65 μ̂ = -0.6
Negative (n=8419) Positive (n=486)
P < 0.0001 ***
μ̂ = 0.14 μ̂ = 1.94
Negative (n=4250) Positive (n=85)
P = 0.0287 *
μ̂ = -0.12 μ̂ = 0.4
Negative (n=58430) Positive (n=553)
P = 0.0018 **
μ̂ = -0.0 μ̂ = 0.67
Negative (n=465) Positive (n=1310)
P = 0.5118 ns
μ̂ = 0.29 μ̂ = 0.47
Negative (n=18213) Positive (n=615)
P = 0.0462 *
μ̂ = 2.91 μ̂ = 3.59
Negative (n=11419) Positive (n=486)
P = 0.5803 ns
μ̂ = -0.22 μ̂ = 0.3
Negative (n=1423) Positive (n=85)
P = 0.4999 ns
μ̂ = 0.3 μ̂ = 0.16
Negative (n=58430) Positive (n=553)
P = 0.0021 **
μ̂ = 0.0 μ̂ = 0.9
Negative (n=465) Positive (n=1310)
P = 0.0230 *
μ̂ = 0.07 μ̂ = 0.57
Negative (n=18213) Positive (n=615)
P = 0.0148 *
μ̂ = -1.31 μ̂ = -0.72
Negative (n=11419) Positive (n=486)
P = 0.0009 ***
μ̂ = 0.09 μ̂ = 1.74
Negative (n=4250) Positive (n=85)
P = 0.1301 ns
μ̂ = -0.19 μ̂ = -0.55
Negative (n=31993) Positive (n=553)
P = 0.4023 ns
μ̂ = -0.0 μ̂ = 0.31
Negative (n=465) Positive (n=1310)

Supplementary Fig. 6. Breast age gap differences between healthy and breast cancer groups for each dataset based on each specific dataset-trained Mammo-AGE model. The figure illustrates the differences in the breast age gap between healthy individuals and those diagnosed with breast cancer across various training and testing datasets. Each row corresponds to a different training dataset, while each column corresponds to a different testing dataset. We retrained our Mammo-AGE model (using ResNet-18 with image size of 1024 × 512) separately using each dataset (Inhouse, RSNA, VinDr, EMBED) from scratch. For each retrained model, the breast age gap in the breast cancer group tends to be higher than in the healthy group across all testing datasets. This consistent trend across all training datasets highlights the robustness and generalizability of the breast age gap as a biomarker for breast cancer risk. The models were retrained separately on each dataset (Inhouse, RSNA, VinDr, EMBED) and subsequently tested on all datasets to assess the robustness and generalizability of the breast age gap as an indicator of breast cancer. Two-sided ANCOVA tests are utilized and adjusted for the chronological age and breast density (Statistical significance: ns: Not significant; *: $P < 0.05$; **: $P < 0.01$; ***: $P < 0.001$). Box plots show the median (center line), the 25th and 75th percentiles (box), and whiskers extending to data within 1.5 times the interquartile range.

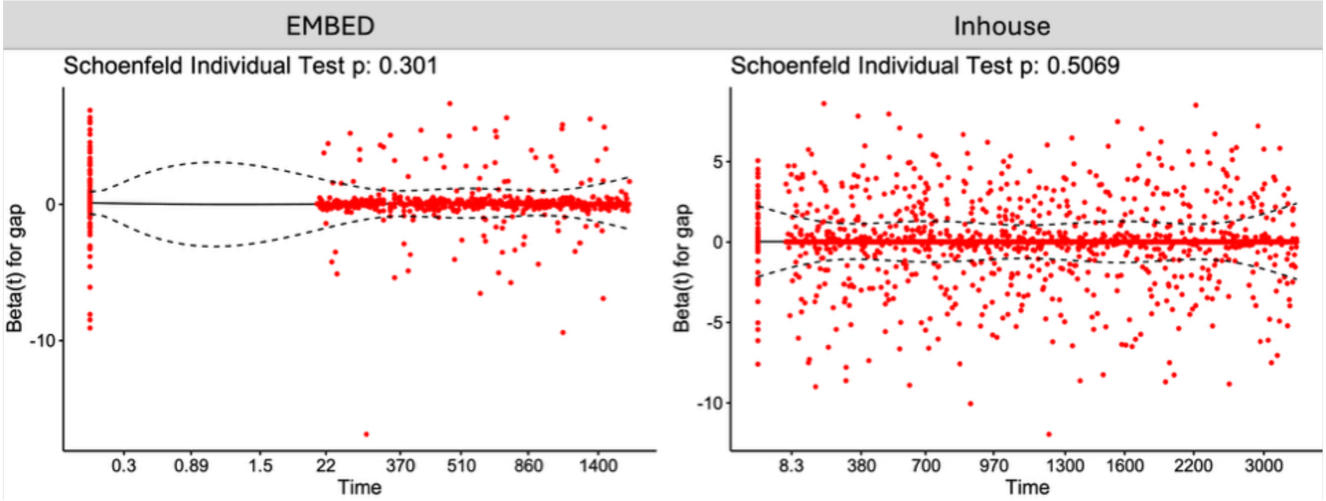

Supplementary Fig. 7. Association between the breast age gap and future breast cancer using Cox proportional hazards regression models. Estimates of the time-dependent coefficients of the models adjusted for chronological age and breast density for outcomes on two datasets. The p-values corresponded to the chi-squared test on the correlation between the Schoenfeld residuals and time.

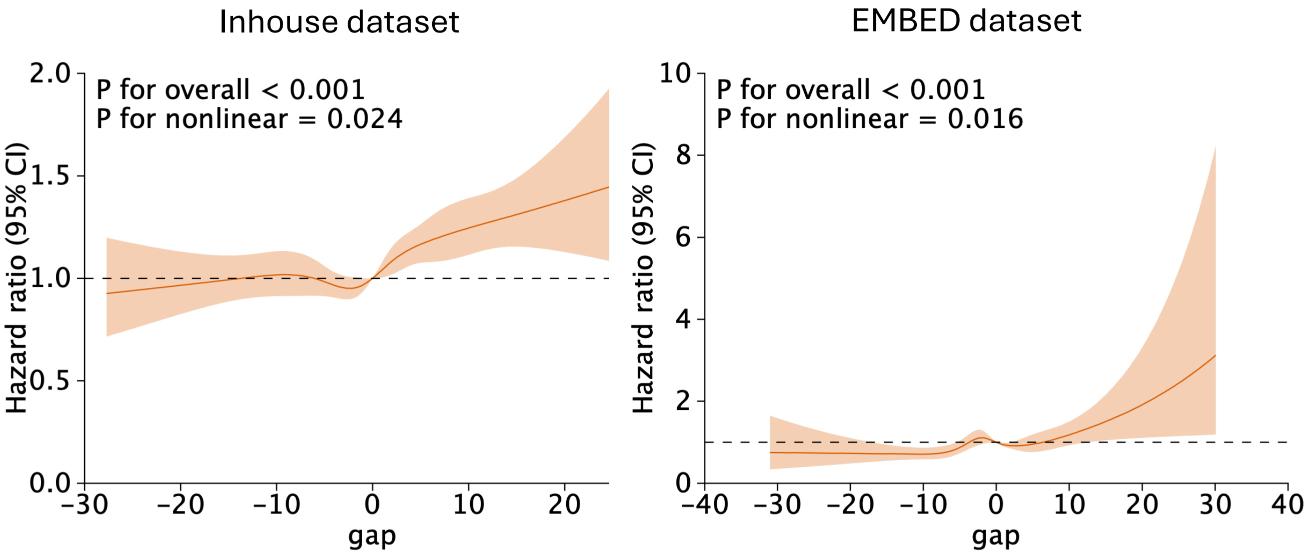

Supplementary Fig. 8. Association between breast age gap (predicted breast age - chronological age) and 10-year (inhouse dataset) or 5-year (EMBED dataset) breast cancer risk using restricted cubic spline regression models. HRs for future breast cancer events according to the breast age gap (predicted breast age - chronological age) adjusted for age and density. In this plot, models were fitted by a restricted cubic spline Cox proportional hazards regression model, and the model was conducted with 6 knots at the 5th, 23th, 41th, 59th, 77th, and 95th percentiles of gap (reference is 0). Solid lines indicate HRs, and shadow shape indicate 95% CIs. HR, hazard ratio; CI, confidence interval. Evidence of an overall and non-linear association between breast age gap and breast cancer risk were observed (Inhouse dataset: $P_{overall}<0.001$; $P_{non\text{-}linear}=0.024$; EMBED dataset: $P_{overall}<0.001$; $P_{non\text{-}linear}=0.016$).

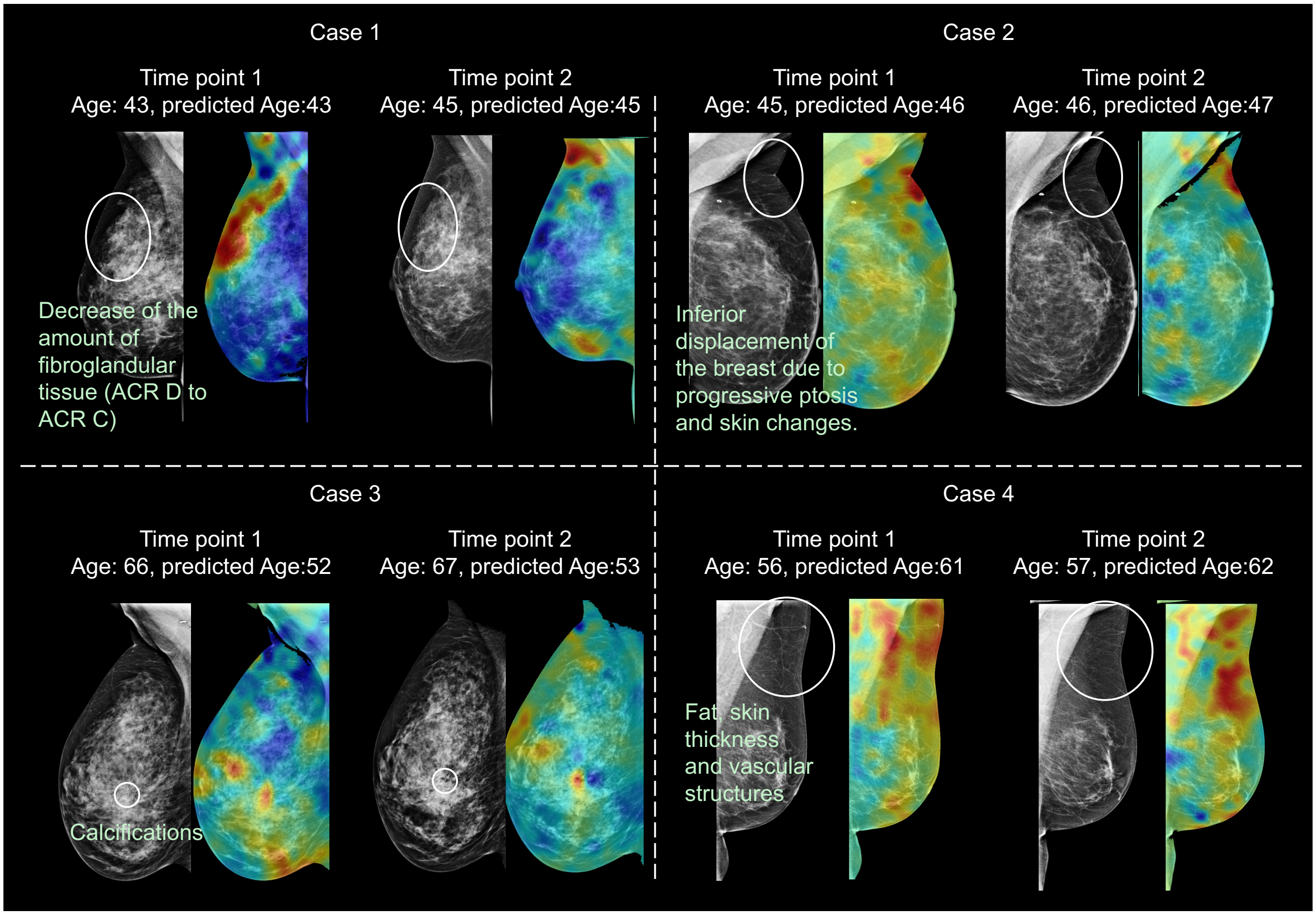

Supplementary Fig. 9. Saliency maps based on longitudinal mammogram examinations for each woman. Mammo-AGE consistently focuses on specific anatomical regions rather than randomly scanning the entire breast. These include fibroglandular tissue, skin thickness, calcifications, masses, and vascular structures, which have been evidenced by previous breast morphology studies that are associated with age-related breast changes [4]. For instance, with increasing age, the epidermis of the female breast continues to thin, the elasticity of the mammary gland decreases, and the mammary gland matrix undergoes ptosis as it is replaced by fatty tissue. Importantly, the model could learn the consistent inherent aging pattern features for each woman over time. These suggest that the model is consistently capturing biologically meaningful patterns from these informative areas.

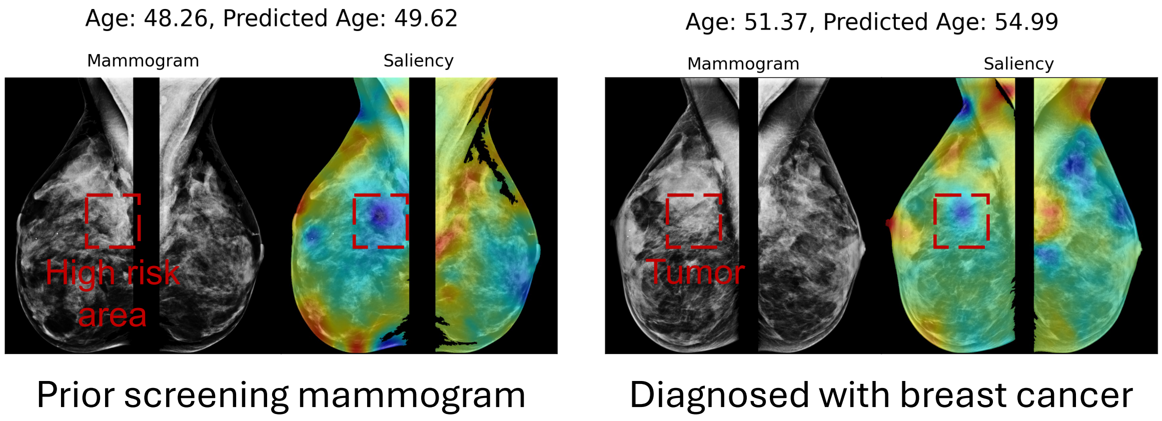

Supplementary Fig. 10. Longitudinal saliency map analysis of the breast age prediction model. Red areas indicate regions important for accurate age estimation. Blue areas indicate abnormal regions that result in higher prediction deviations. Longitudinal analysis shows that the blue area is biologically abnormal and has a high risk of developing breast cancer in the future.

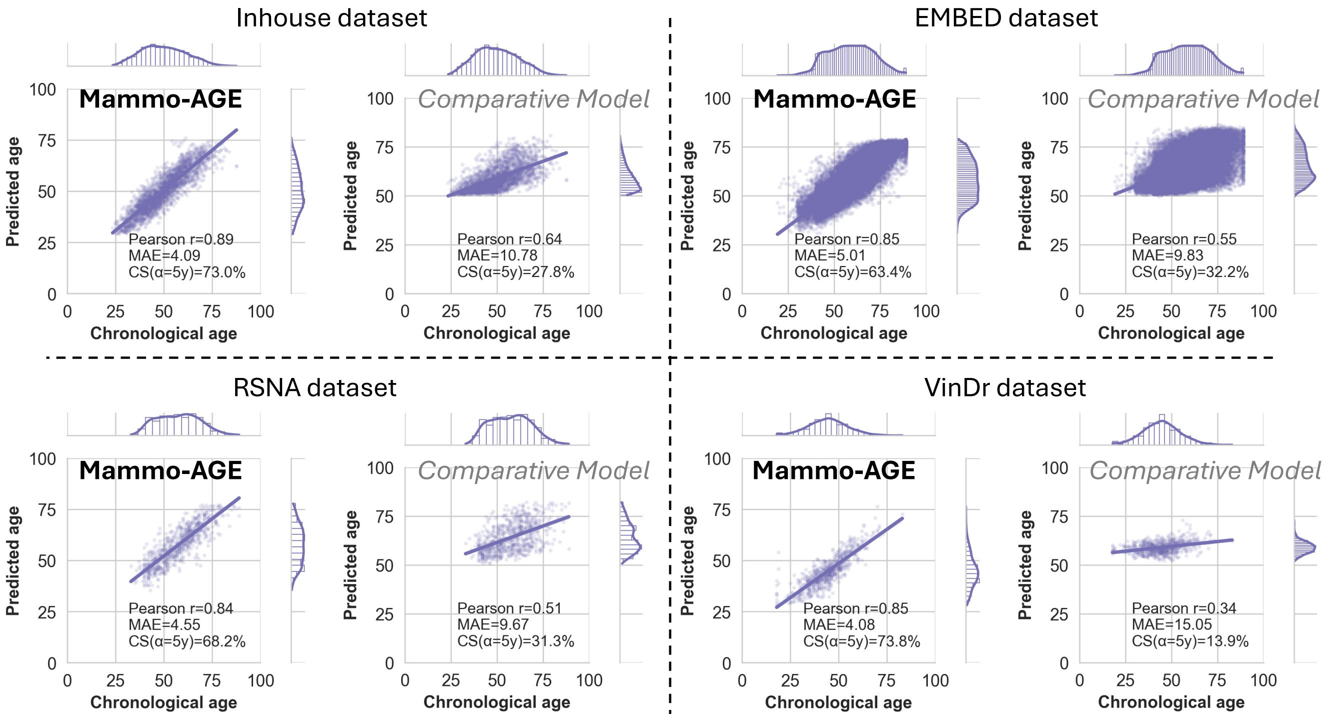

Supplementary Fig. 11. Comparison results of age prediction with other comparative model [5]. The comparative model aims to predict breast cancer risk, with age prediction as an auxiliary task. We have implemented the comparison experiments across multiple datasets. Mammo-AGE consistently outperformed it, achieving a lower MAE and also higher correlation and accuracy, demonstrating its superior ability to capture biologically relevant breast aging features. Unlike comparative model, which predicts age in coarse bins (six age groups: 40-100 years), Mammo-AGE estimates continuous biological age, allowing for a more precise and clinically meaningful assessment of breast tissue aging.

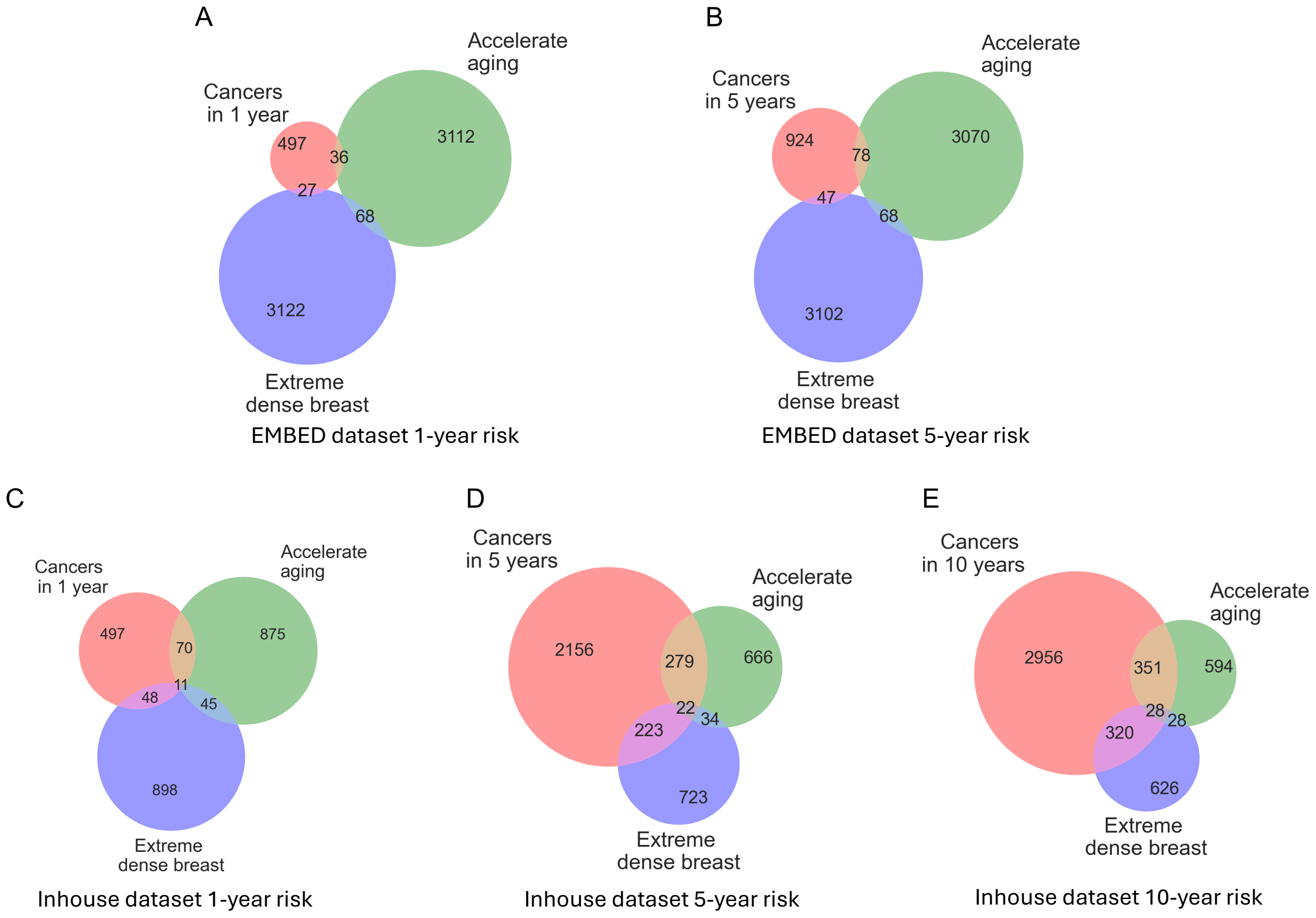

Supplementary Fig. 12. Venn diagrams comparing true positive cancer detection between the Mammo-AGE model (orange) and women with extremely dense breasts (purple), based on the selection criteria used in the DENSE trial [6]. Red areas represent cancers diagnosed within 1-, 5-, or 10-year follow-up periods in the inhouse and EMBED datasets. Under equivalent high-risk selection thresholds (i.e., selecting the same number of women), the Mammo-AGE model identifies a greater number of cancers.

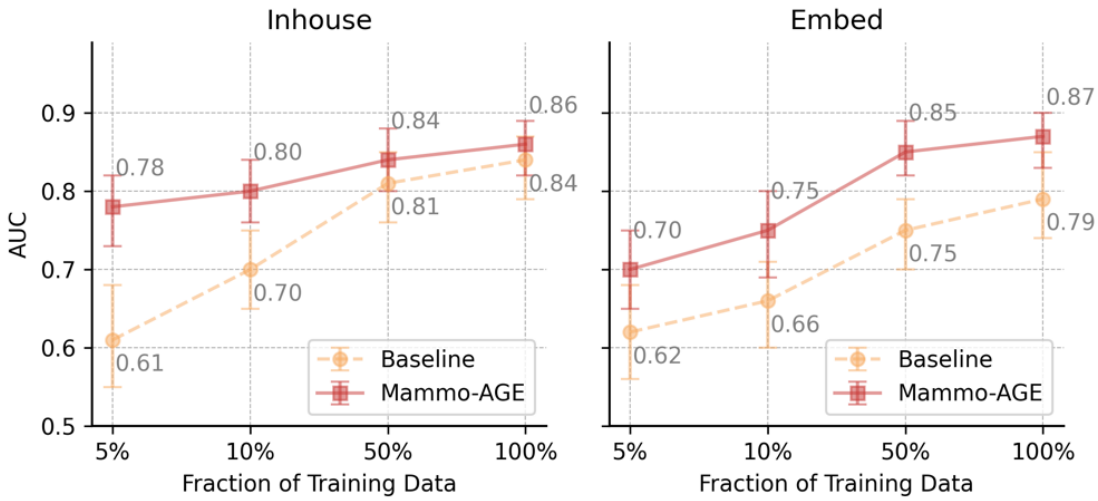

Supplementary Fig. 13. AUC results with different fractions of training data during finetuning on the task of breast cancer classification. Mammo-AGE consistently outperformed training from scratch (i.e., baseline) on both inhouse (n = 2,208), EMBED (n = 1,978) datasets. AUC: Area Under Receiver Operating Characteristic Curve; The error bars represent the 95% confidence intervals (CI) of AUC metrics, estimated from 1,000 bootstrap samples for each measure. Center values represent the mean performance across bootstrap replicates.

Supplementary Table 1. Results of Mammo-AGE models' performance with ensemble and different backbones on the combined dataset using five-fold cross-validation.

			Fold 1	Δ	Fold 2	Δ	Fold 3	Δ	Fold 4	Δ	Fold 5	Δ	Mean±SD	Δ
**Ours ensemble Mammo-AGE**		MAE	4.230	-	4.150	-	4.167	-	4.161	-	4.164	-	4.174±0.032	-
		Pearson Correlation *(r)*	0.890	-	0.892	-	0.892	-	0.892	-	0.892	-	0.891±0.001	-
		CS % (α=5 year)	71.073	-	73.132	-	72.369	-	72.674	-	71.886	-	72.7%±0.8%	-
Different backbones (1536×768)	Ours Mammo-AGE (ResNet-18)	MAE	4.738	**-0.508**	4.631	**-0.481**	4.615	**-0.448**	4.645	**-0.484**	4.711	**-0.547**	4.668±0.053	**-0.493**
		Pearson Correlation *(r)*	0.861	**-0.029**	0.866	**-0.026**	0.868	**-0.024**	0.866	**-0.026**	0.862	**-0.030**	0.864±0.003	**-0.027**
		CS % (α=5 year)	66.0	**-5.1**	67.4	**-5.8**	67.1	**-5.3**	67.4	**-5.3**	66.5	**-5.4**	66.9%±0.6%	**-5.358**
	Ours Mammo-AGE (ResNet-50)	MAE	4.877	**-0.647**	4.790	**-0.640**	4.956	**-0.789**	4.943	**-0.782**	4.772	**-0.608**	4.868±0.085	**-0.693**
		Pearson Correlation (r)	0.853	**-0.037**	0.854	**-0.038**	0.847	**-0.044**	0.850	**-0.042**	0.854	**-0.038**	0.852±0.003	**-0.040**
		CS % (α=5 year)	65.2	**-5.9**	65.3	**-7.8**	64.0	**-8.4**	63.8	**-8.9**	65.1	**-6.8**	64.7%±0.7%	**-7.565**
	Ours Mammo-AGE (EfficcientNet-B0)	MAE	4.676	**-0.446**	4.675	**-0.525**	4.701	**-0.534**	4.644	**-0.483**	4.800	**-0.636**	4.699±0.060	**-0.525**
		Pearson Correlation *(r)*	0.867	**-0.023**	0.862	**-0.030**	0.861	**-0.031**	0.853	**-0.039**	0.861	**-0.031**	0.861±0.005	**-0.031**
		CS % (α=5 year)	65.9	**-5.2**	67.5	**-5.6**	67.1	**-5.3**	67.8	**-4.9**	65.5	**-6.4**	66.8%±1.0%	**-5.450**
	Ours Mammo-AGE (DenseNet-121)	MAE	4.532	**-0.302**	4.632	**-0.482**	4.620	**-0.453**	4.584	**-0.423**	4.586	**-0.422**	4.591±0.039	**-0.416**
		Pearson Correlation *(r)*	0.870	**-0.020**	0.871	**-0.021**	0.868	**-0.024**	0.867	**-0.025**	0.868	**-0.024**	0.869±0.002	**-0.023**
		CS % (α=5 year)	68.0	**-3.1**	66.7	**-6.4**	66.6	**-5.8**	67.5	**-5.1**	67.0	**-4.9**	67.2%±0.6%	**-5.053**
	Ours Mammo-AGE (Convnext-Tiny)	MAE	4.423	**-0.193**	4.307	**-0.157**	4.255	**-0.088**	4.290	**-0.129**	4.287	**-0.123**	4.312±0.065	**-0.138**
		Pearson Correlation (r)	0.879	**-0.010**	0.883	**-0.009**	0.887	**-0.005**	0.887	**-0.006**	0.885	**-0.006**	0.884±0.003	**-0.007**
		CS % (α=5 year)	69.2	**-1.9**	70.8	**-2.4**	71.7	**-0.7**	70.7	**-2.0**	70.7	**-1.2**	70.6%±0.9%	**-1.632**

Supplementary Table 2. Results of the ablation studies on the combined dataset using five-fold cross-validation. The first ablation study compares model performance across different modules. The second ablation study examines the model performance with different image sizes. The default backbone is ResNet-18.

			Fold 1	Δ	Fold 2	Δ	Fold 3	Δ	Fold 4	Δ	Fold 5	Δ	Mean±SD	Δ
	Ours Mammo-AGE (ResNet-18) (1024×512) (w/ all)	MAE	4.848	-	4.670	-	4.695	-	4.807	-	4.662	-	4.736±0.076	-
		Pearson Correlation *(r)*	0.853	-	0.858	-	0.859	-	0.850	-	0.858	-	0.855±0.004	-
		CS % (α=5 year)	65.2	-	67.2	-	66.5	-	66.5	-	67.1	-	66.5%±0.7%	-
Ablation different module (ResNet18) (1024×512)	Ours (w/o CI Transformer)	MAE	4.838	+0.010	4.911	**-0.241**	4.879	**-0.184**	4.853	**-0.046**	4.887	**-0.225**	4.874±0.026	**-0.137**
		Pearson Correlation *(r)*	0.847	**-0.006**	0.844	**-0.013**	0.855	**-0.005**	0.852	+0.002	0.852	**-0.006**	0.850±0.004	**-0.006**
		CS % (α=5 year)	66.1	+0.988	65.1	**-2.129**	64.9	**-1.571**	65.3	**-1.191**	64.7	**-2.382**	65.2%±0.5%	**-1.257**
	Ours (w/o POE)	MAE	4.781	+0.067	4.842	**-0.172**	4.734	**-0.040**	4.814	**-0.008**	4.749	**-0.086**	4.784±0.040	**-0.048**
		Pearson Correlation *(r)*	0.852	**-0.000**	0.847	**-0.010**	0.859	**-0.000**	0.853	+0.003	0.854	**-0.004**	0.853±0.004	**-0.002**
		CS % (α=5 year)	65.3	+0.177	65.5	**-1.749**	66.3	**-0.203**	65.4	**-1.166**	65.9	**-1.191**	65.7%±0.4%	**-0.826**
	Ours (w/o MVL)	MAE	4.911	**-0.063**	4.771	**-0.100**	4.779	**-0.085**	4.855	**-0.048**	4.782	**-0.120**	4.820±0.055	**-0.083**
		Pearson Correlation *(r)*	0.848	**-0.004**	0.850	**-0.008**	0.851	**-0.008**	0.848	**-0.002**	0.848	**-0.010**	0.849±0.001	**-0.006**
		CS % (α=5 year)	64.3	**-0.811**	67.1	**-0.152**	66.6	+0.127	64.9	**-1.673**	66.8	**-0.355**	65.9%±1.1%	**-0.573**
	Ours (w/o Multi-Task)	MAE	4.807	+0.041	4.949	**-0.279**	4.809	**-0.114**	4.693	+0.113	4.763	**-0.101**	4.778±0.023	**-0.068**
		Pearson Correlation (r)	0.857	+0.004	0.850	**-0.007**	0.852	**-0.007**	0.858	+0.008	0.853	**-0.005**	0.863±0.003	**-0.001**
		CS % (α=5 year)	64.9	**-0.228**	63.9	**-3.269**	65.9	**-0.532**	67.4	+0.836	66.4	**-0.710**	66.2%±0.4%	**-0.781**
Different image size Mammo-AGE (ResNet-18)	Ours (2048×1024)	MAE	5.058	**-0.209**	5.177	**-0.507**	5.394	**-0.700**	5.284	**-0.477**	5.100	**-0.437**	5.202±0.123	**-0.466**
		Pearson Correlation (r)	0.836	**-0.016**	0.835	**-0.023**	0.821	**-0.038**	0.832	**-0.018**	0.837	**-0.021**	0.832±0.006	**-0.023**
		CS % (α=5 year)	63.0	**-2.114**	62.5	**-4.752**	60.3	**-6.152**	60.8	**-5.771**	62.2	**-4.905**	61.8%±1.0%	**-4.739**
	**(Selected) Ours (1536×768)**	MAE	4.738	**+0.111**	4.631	**+0.039**	4.615	**+0.080**	4.645	**+0.162**	4.711	**-0.049**	4.668±0.053	**+0.068**
		Pearson Correlation (r)	0.861	**+0.008**	0.866	**+0.008**	0.868	**+0.009**	0.866	**+0.016**	0.862	**+0.004**	0.864±0.003	**+0.009**
		CS % (α=5 year)	66.0	**+0.834**	67.4	**+0.154**	67.1	**+0.635**	67.4	**+0.864**	66.5	**-0.634**	66.9%±0.6%	**+0.371**
	Ours (512×256)	MAE	5.068	**-0.220**	5.016	**-0.346**	4.990	**-0.295**	4.973	**-0.167**	5.046	**-0.383**	5.019±0.035	**-0.282**
		Pearson Correlation (r)	0.833	**-0.019**	0.835	**-0.023**	0.837	**-0.022**	0.841	**-0.009**	0.836	**-0.022**	0.836±0.002	**-0.019**
		CS % (α=5 year)	64.0	**-1.166**	64.3	**-2.889**	64.4	**-2.078**	63.8	**-2.762**	64.0	**-3.142**	64.1%±0.2%	**-2.408**
	Ours (256×128)	MAE	5.388	**-0.540**	5.370	**-0.700**	5.448	**-0.753**	5.387	**-0.580**	5.421	**-0.759**	5.403±0.028	**-0.666**
		Pearson Correlation (r)	0.806	**-0.046**	0.804	**-0.054**	0.798	**-0.061**	0.805	**-0.045**	0.803	**-0.055**	0.803±0.003	**-0.052**
		CS % (α=5 year)	61.1	**-4.029**	61.4	**-5.854**	60.0	**-6.462**	61.5	**-5.068**	60.7	**-6.412**	60.9%±0.5%	**-5.565**

Supplementary Table 3. Association between chronological age / predicted breast age with future breast cancer using Cox proportional hazards regression models. Note: In calculating hazard ratios, breast ages were predicted from mammograms and have not been adjusted for chronological age. Therefore, the results indicate that breast age may serve as a similar risk factor for breast cancer as chronological age. *P*-values were calculated using two-sided Wald tests.

	Years,				Model 1 (Unadj.)		Model 2 (ACR-adj.)	
Chronological age	mean±SD	N	Events	Inc.	HR (95%CI)	*P* value	HR (95%CI)	*P* value
Inhouse BC events (10Ys)	53.08±11.63	10,392	3,656	35.2	-	-	-	-
Age, per one year	-	-	-	-	1.032 (1.029-1.035)	<2e-16	1.033 (1.030-1.036)	< 2e-16
1st quartile (<44.1)	38.49±4.20	2,598	614	23.6	1.000 (Reference)	-	1.000 (Reference)	-
2nd quartile (44.1-52.6)	48.38±2.49	2,599	793	30.5	1.361 (1.225-1.513)	9.51e-09	1.372 (1.235-1.525)	4.67e-09
3rd quartile (52.6-61.9)	56.97±2.69	2,597	973	37.5	1.746 (1.578-1.931)	< 2e-16	1.789 (1.615-1.982)	< 2e-16
4th quartile (>61.9)	68.46±4.85	2,598	1,276	49.1	2.570 (2.334-2.830)	< 2e-16	2.655 (2.405-2.931)	< 2e-16
HR trend, *P* for trend	-	-	-	-	1.366 (1.326-1.408)	< 2e-16	1.382 (1.340-1.426)	< 2e-16
	Years,				Model 3 (Unadj.)		Model 4 (ACR-adj.)	
Breast AGE	mean±SD	N	Events	Inc.	HR (95%CI)	*P* value	HR (95%CI)	*P* value
Inhouse BC events (10Ys)	53.45±12.51	10,392	3,656	35.2	-	-	-	-
Age, per one year	-	-	-	-	1.030 (1.027-1.032)	<2e-16	1.031 (1.028-1.034)	< 2e-16
1st quartile (<43.6)	37.50±4.60	2,598	607	23.4	1.000 (Reference)	-	1.000 (Reference)	-
2nd quartile (43.6-53.3)	48.39±2.82	2,598	776	29.9	1.343 (1.208-1.494)	5.21e-08	1.365 (1.227-1.518)	1.11e-08
3rd quartile (53.3-63.2)	58.12±2.81	2,598	1,020	39.3	1.883 (1.703-2.082)	< 2e-16	1.934 (1.746-2.143)	< 2e-16
4th quartile (>63.2)	69.79±4.49	2,598	1,253	48.2	2.547 (2.311-2.806)	< 2e-16	2.625 (2.375-2.901)	< 2e-16
HR trend, *P* for trend	-	-	-	-	1.369 (1.329-1.411)	< 2e-16	1.383 (1.340-1.426)	< 2e-16
	Years,				Model 1 (Unadj.)		Model 2 (ACR-adj.)	
Chronological age	mean±SD	N	Events	Inc.	HR (95%CI)	*P* value	HR (95%CI)	*P* value
EMBED BC events (5Ys)	58.95±11.94	58,983	1,029	3.49	-	-	-	-
Age gap, per one age (Ys)	-	-	-	-	1.030 (1.025-1.036)	<2e-16	1.033 (1.028-1.038)	<2e-16
1st quartile (<49.5)	43.80±4.15	14,751	164	2.22	1.000 (Reference)	-	1.000 (Reference)	-
2nd quartile (49.5-58.8)	54.18±2.65	14,741	206	2.79	1.361 (1.225-1.513)	0.028	1.306 (1.062-1.606)	9.92e-03
3rd quartile (58.8-65.5)	63.33±2.65	14,751	240	3.25	1.467 (1.203-1.789)	1.57e-04	1.601 (1.308-1.960)	8.25e-06
4th quartile (>65.5)	74.49±5.13	14,740	419	5.69	2.575 (2.150-3.085)	< 2e-16	2.838 (2.358-3.415)	< 2e-16
HR trend, *P* for trend	-	-	-	-	1.380 (1.304-1.461)	<2e-16	1.427 (1.346-1.513)	< 2e-16
	Years,				Model 3 (Unadj.)		Model 4 (ACR-adj.)	
Breast AGE	mean±SD	N	Events	Inc.	HR (95%CI)	*P* value	HR (95%CI)	*P* value
EMBED BC events (5Ys)	57.57±9.51	58,983	1,029	3.49				
Age, per one year	-	-	-	-	1.038 (1.031-1.045)	<2e-16	1.042 (1.035-1.049)	<2e-16
1st quartile (<49.7)	45.43±3.01	14,746	176	2.39	1.000 (Reference)	-	1.000 (Reference)	-
2nd quartile (49.7-57.4)	53.51±2.24	14,746	192	2.60	1.091 (0.889-1.339)	0.403	1.149 (0.936-1.412)	0.011
3rd quartile (57.4-65.2)	61.31±2.24	14,745	258	3.50	1.469 (1.213-1.780)	8.28e-05	1.596 (1.314-1.939)	2.52e-06
4th quartile (>65.2)	70.03±3.07	14,746	403	5.47	2.305 (1.931-2.752)	< 2e-16	2.543 (2.120-3.051)	< 2e-16
HR trend, *P* for trend	-	-	-	-	1.350 (1.275-1.429)	<2e-16	1.393 (1.315-1.476)	<2e-16

Inc = incidence per 1,000 person-years; CI = confidence interval; BC =breast cancer; HR = hazard ratio; Unadj. HR = unadjusted HR; ACR-adj. HR =HR adjusted HR on breast density; Breast AGE = deep learning-based breast biological age.

Supplementary Table 4. Comparisons of model performance for different methods. Ours: Mammo-AGE model; SV: single-view-based baseline method. POE: probabilistic ordinal embedding method; MVL: mean-variance loss method; GLT: global-local transformer method; MV: multi-view-based baseline method; MAE: mean absolute error, lower is better; CS: cumulative score based on a threshold of error within five years (α=5y), higher is better. Pearson correlation coefficient and Spearman correlation coefficients between predicted breast age and chronological age are also reported.

		Test N_{sample}	3,946	2,489	750	707	33,040	465
		Age range	18-89	23-87	33-89	18-83	19-89	18-84
Method			Combined Dataset	Inhouse Dataset	RSNA Dataset	VinDr Dataset	EMBED Dataset	CMMD Dataset
Compare with other methods (ResNet18)	Single view	MAE	7.429±0.156	6.988±0.134	8.362±0.300	7.991±0.373	9.741±0.338	7.213±0.354
		Pearson Correlation (r)	0.676±0.017	0.718±0.011	0.591±0.028	0.509±0.031	0.600±0.024	0.438±0.048
		Spearman Correlation (r_s)	0.704±0.017	0.740±0.010	0.602±0.025	0.509±0.021	0.610±0.025	0.400±0.034
		CS (α=5 year)	45.6%±1.0%	47.0%±1.0%	40.4%±1.4%	46.0%±2.1%	36.0%±1.3%	49.4%±2.1%
	POE	MAE	5.689±0.064	5.460±0.025	5.872±0.056	6.303±0.297	6.376±0.030	6.054±0.122
		Pearson Correlation (r)	0.786±0.005	0.799±0.002	0.729±0.005	0.646±0.008	0.748±0.002	0.594±0.012
		Spearman Correlation (r_s)	0.795±0.005	0.809±0.002	0.729±0.006	0.642±0.008	0.752±0.002	0.521±0.006
		CS (α=5 year)	58.1%±0.5%	59.5%±0.3%	56.5%±0.7%	55.1%±2.3%	52.8%±0.2%	54.1%±1.3%
	MVL	MAE	5.578±0.037	5.406±0.030	5.767±0.043	5.985±0.094	6.313±0.024	6.005±0.079
		Pearson Correlation (r)	0.796±0.004	0.805±0.004	0.741±0.005	0.658±0.008	0.759±0.001	0.595±0.006
		Spearman Correlation (r_s)	0.804±0.005	0.814±0.003	0.741±0.006	0.656±0.011	0.763±0.001	0.528±0.007
		CS (α=5 year)	58.9%±0.2%	59.7%±0.2%	57.5%±0.6%	57.7%±0.9%	53.1%±0.2%	54.1%±1.3%
	GLT	MAE	5.629±0.036	5.491±0.021	5.806±0.018	5.927±0.133	6.349±0.047	6.095±0.144
		Pearson Correlation (r)	0.793±0.002	0.800±0.001	0.738±0.002	0.661±0.008	0.753±0.002	0.598±0.016
		Spearman Correlation (r_s)	0.803±0.002	0.810±0.001	0.737±0.002	0.656±0.011	0.758±0.002	0.526±0.011
		CS (α=5 year)	58.3%±0.3%	58.8%±0.3%	56.9%±0.6%	58.0%±0.7%	52.8%±0.4%	54.2%±1.1%
	Multi-view	MAE	5.079±0.035	4.841±0.051	5.514±0.083	5.456±0.141	5.944±0.112	6.192±0.136
		Pearson Correlation (r)	0.829±0.004	0.845±0.006	0.758±0.007	0.680±0.011	0.785±0.004	0.602±0.018
		Spearman Correlation (r_s)	0.838±0.004	0.852±0.006	0.761±0.008	0.689±0.010	0.792±0.004	0.531±0.022
		CS (α=5 year)	63.7%±0.2%	65.0%±0.4%	60.1%±0.6%	62.7%±1.3%	55.9%±1.1%	53.0%±1.7%
Ours ensembled Mammo-AGE		MAE	4.174±0.028	4.090±0.047	4.548±0.022	4.075±0.010	5.010±0.040	6.103±0.222
		Pearson Correlation (r)	0.891±0.001	0.892±0.002	0.844±0.001	0.847±0.002	0.855±0.002	0.705±0.009
		Spearman Correlation (r_s)	0.896±0.001	0.898±0.002	0.849±0.002	0.843±0.003	0.861±0.002	0.634±0.009
		CS (α=5 year)	72.2%±0.7%	73.0%±0.8%	68.2%±0.7%	73.8%±0.8%	63.4%±0.5%	54.8%±1.5%

Supplementary Table 5. Ablation study on integrating predicted breast density into the embedding layer for age prediction, evaluated using five-fold cross-validation on the combined dataset. We compare the performance of Mammo-AGE models with and without incorporating predicted density across different backbones and a final ensemble model. This adjustment indeed led to performance improvements, reducing MAE by (Δ=0.002-0.077) across different backbone-based models and our ensemble Mammo-AGE model.

Model	MAE	Pearson Correlation (r)	CS % (α=5 year)
Mammo-AGE (Ensemble)	4.174±0.032	0.891±0.001	72.7%±0.8%
(w/o ACR)	4.301±0.030	0.881±0.002	71.4%±0.5%
Mammo-AGE (ResNet-18)	4.728±0.034	0.855±0.003	66.5%±0.4%
(w/o ACR)	4.736±0.076	0.855±0.004	66.5%±0.7%
Mammo-AGE (ResNet-50)	4.875±0.044	0.848±0.003	64.6%±0.5%
(w/o ACR)	4.947±0.074	0.845±0.004	64.4%±0.4%
Mammo-AGE (EfficientNet-B0)	4.512±0.032	0.866±0.004	69.1%±0.2%
(w/o ACR)	4.589±0.020	0.855±0.004	66.5%±0.7%
Mammo-AGE (DenseNet-121)	4.688±0.027	0.859±0.002	66.6%±0.2%
(w/o ACR)	4.690±0.054	0.861±0.004	66.6%±0.6%
Mammo-AGE (Convnext-Tiny)	4.526±0.046	0.864±0.005	68.8%±0.4%
(w/o ACR)	4.579±0.079	0.865±0.004	68.1%±0.4%

Supplementary Table 6. Accuracy of density prediction on each dataset.

Dataset	AUC (Four classes)	ACC (Four classes)	AUC (Two classes)	ACC (Two classes)
Inhouse	0.790±0.005	0.677±0.005	0.877±0.007	0.811±0.003
RSNA	0.792±0.009	0.623±0.016	0.888±0.008	0.785±0.018
VinDr	0.780±0.017	0.799±0.015	0.949±0.007	0.938±0.008
EMBED	0.783±0.006	0.614±0.012	0.898±0.003	0.792±0.009

Supplementary Table 7. Breast age gap differences between healthy women and breast cancer patients, and statistical analysis with and without adjustment by age and breast density. Unadjusted *P*-values: obtained from two-sided t-tests. Adjusted *P*-values: obtained from two-sided ANCOVA tests, adjusted for chronological age and breast density (Statistical significance: *: $P < 0.05$; **: $P < 0.01$; ***: $P < 0.001$).

Dataset	Breast age gap (Mean±STD) Healthy population	Breast age gap (Mean±STD) Breast cancer patients	Un-adjusted *P*-values	Adjusted *P*-values
Inhouse	0.373±4.546	0.819±4.751	0.024 *	0.017 *
RSNA	-1.883±4.851	-0.951±5.031	7.92e-5 ***	2.61e-5 ***
VinDr	-0.059±4.149	0.888±4.225	0.048 *	0.033 *
EMBED	0.073±5.026	0.489±4.885	0.047 *	0.039 *
CMMD	0.383±4.732	0.887±4.773	0.049 *	0.049 *

Supplementary Table 8. Odds ratios analysis for breast cancer based on breast density and breast age gap. *P*-values are calculated using two-sided Wald tests.

	Inhouse 10-year risk		EMBED 5-year risk	
	Odds ratios	P-value	Odds ratios	P-value
Breast density	1.060 (1.018-1.104)	0.005	1.069 (1.005-1.136)	0.03
Breast age gap (per year)	1.070 (1.028-1.114)	0.0010	1.114 (1.048-1.183)	0.0005

Supplementary Table 9. Odds ratios and diagnostic age of each risk group. Decelerated aging group: gap<(Mean-1.96SD); Medium aging group: (Mean-1.96SD, Mean+1.96SD); and Accelerated aging group: gap>(Mean+1.96SD). *P*-values are calculated using two-sided Wald tests.

Risk Group	Odds Ratios (95% CI)	*P* Value	Avg. Diagnostic Age (95% CI)
Per SD Increase in Breast Age Gap	1.116 (1.036-1.203)	0.0040	63 (38-89)
Decelerated aging group	Reference	-	72 (47-98)
Medium aging group	1.927 (0.957-3.878)	0.0661	63 (38-89]
Accelerated aging group	2.966 (1.341-6.563)	0.0073	62 (47-80)

Reference

1. He, S., Grant, P. E. & Ou, Y. Global-Local Transformer for Brain Age Estimation. *IEEE Trans. Med. Imag.* **41**, 213–224 (2021).
2. Pan, H., Han, H., Shan, S. & Chen, X. Mean-variance loss for deep age estimation from a face. in *Proceedings of the IEEE conference on computer vision and pattern recognition* 5285–5294 (2018).
3. Li, W., Huang, X., Lu, J., Feng, J. & Zhou, J. Learning probabilistic ordinal embeddings for uncertainty-aware regression. *Proceedings of the IEEE Computer Society Conference on Computer Vision and Pattern Recognition* 13891–13900 (2021) doi:10.1109/CVPR46437.2021.01368.
4. Lin, J. *et al.* Changes in the mammary gland during aging and its links with breast diseases. *Acta Biochim. Biophys. Sin.* **55**, 1001–1019 (2023).
5. Adam Yala, Peter G Mikhael 1 , Fredrik Strand 2, 3 , Gigin Lin 4 , Kevin Smith 5, 6 , Yung-Liang Wan 4 , Leslie Lamb 7 , Kevin Hughes 8 , Constance Lehman† 7, R. B. Towards Robust Mammography-Based Models for Breast Cancer Risk. *SCIENCE TRANSLATIONAL MEDICINE* **13**, 1–51 (2021).
6. Bakker, M. F. *et al.* Supplemental MRI screening for women with extremely dense breast tissue. *N. Engl. J. Med.* **381**, 2091–2102 (2019).
7. Dench, E. *et al.* Measurement challenge: Protocol for international case–control comparison of mammographic measures that predict breast cancer risk. *BMJ Open* **9**, e031041 (2019).
